# Insights from the Evolution of Coagulation: A New Perspective on Anti-Inflammatory Strategies in the ICU—Focus on the Contact Activation System

**DOI:** 10.3390/biomedicines13112726

**Published:** 2025-11-06

**Authors:** Ruihua Wang, Feng Zhu

**Affiliations:** Department of Critical Care Medicine, Shanghai East Hospital, School of Medicine, Tongji University, Shanghai 200120, China; 1426187@163.com

**Keywords:** coagulation factor XII, contact activation system, kallikrein–kinin system, immunothrombosis, sepsis

## Abstract

This review reappraises the anti-inflammatory potential of the contact activation system (CAS) in intensive care through an evolutionary lens. The authors propose that coagulation factor XII (FXII) and related components evolved in terrestrial animals as a “foreign-surface sensing–immunothrombosis” module, helping to explain the minimal bleeding phenotype of FXII deficiency and the secondary loss of F12 in marine mammals. CAS shares components with the kallikrein–kinin system (KKS): alpha-coagulation factor XIIa (α-FXIIa) drives coagulation factor XI (FXI) activation to amplify coagulation, whereas betacoagulation factor XIIa (β-FXIIa) activates the KKS to generate bradykinin, promoting vasodilation and vascular leak. Beyond proteolysis, zymogen FXII signals via urokinase-type plasminogen activator receptor (uPAR) to induce neutrophil extracellular trap formation (NETosis), thereby amplifying immunothrombosis. Clinically, the relevance spans sepsis and extracorporeal organ support: pathogens can hijack CAS/KKS to facilitate invasion, and artificial surfaces such as extracorporeal membrane oxygenation (ECMO) circuits chronically trigger contact activation. In animal models, selective inhibition of FXII/FXI prolongs circuit life and attenuates pulmonary edema and inflammation without materially increasing bleeding. The review also catalogs “non-coagulation” roles of CAS members: Activated coagulation factor XI (FXIa) modulates endothelial permeability and smooth-muscle migration, and the FXII heavy chain exhibits direct antimicrobial activity—underscoring CAS as a nexus for coagulation, inflammation, and host defense. Overall, CAS inhibitors may couple “safe anticoagulation” with “cascade-level anti-inflammation,” offering a testable translational path for organ protection in the ICU alongside infection control and informing combined, precision strategies for anticoagulation and anti-inflammatory therapy.

## 1. Evolution of the Coagulation System: From Hemostasis to Non-Self Recognition

### 1.1. Coagulation in Early-Diverging Animals

The origins of the coagulation system can be traced back to the earliest marine organisms with circulatory systems, where its primary function was to seal damaged tissues with fibrin clots to prevent fluid loss [1]. This hemostatic role forms the core of the coagulation system. In lower animals, such as lampreys, the coagulation system is relatively simple, relying primarily on the extrinsic pathway: exposure of tissue factor activates thrombin, which catalyzes fibrinogen to form fibrin, sealing wounds [2]. To enhance hemostatic efficiency, an auxiliary pathway mediated by factor IX evolved, further promoting thrombin generation through activation of factor X [3,4]. This dual “extrinsic + factor IX” system was sufficient to address fluid loss in open circulatory systems [5]. In these organisms, blood cells exhibit a high degree of coupling between coagulation and immune functions. For instance, lamprey thrombocytes secrete thrombin-like proteins that not only form clots to seal wounds but also encapsulate invading microbes, forming an immune defense barrier [6]. The open circulatory system allows mutual activation of inflammation and thrombosis, conferring a unique survival advantage [4]. For example, thrombin can activate immune-related signaling pathways, promoting the release of antibacterial proteins. This coagulation-immune symbiosis laid the foundation for the evolution of closed circulatory systems [7,8].

As vertebrates transitioned from open to closed circulatory systems, the complexity of the intravascular environment increased, requiring the coagulation system to address new challenges, such as pathogen invasion and the risk of thrombus formation. This drove the emergence of coagulation factors upstream of factor IX, with coagulation FXII playing a pivotal role in terrestrial adaptation [9,10].

### 1.2. From Land to Sea: FXII’s Role in Non-Self Recognition

The classic APTT assay involves adding kaolin to plasma to trigger the coagulation cascade, with kaolin being a naturally abundant substance—essentially, the soil found ubiquitously on land [11]. The APTT assay mimics the pathological scenario of a terrestrial animal’s wound being exposed to soil containing kaolin. The prolonged APTT in FXII-deficient patients without bleeding tendencies underscores the evolutionary prioritization of FXII in anti-infection defense over hemostasis. This characteristic positions FXII as an ideal anti-inflammatory target: inhibiting FXII can block microthrombus formation and bradykinin (BK) storms without compromising physiological hemostasis [12]. As lower animals evolved closed circulatory systems and marine organisms transitioned to terrestrial environments, they faced a far more complex milieu than seawater. Terrestrial soil is teeming with diverse microorganisms, and injuries from branches, rocks, or predator bites significantly increase the likelihood of harm compared to marine environments [13]. When a blood vessel ruptures, pathogens can enter the bloodstream alongside soil particles. Upon contact with foreign surfaces, coagulation factors trigger the coagulation cascade, generating thrombin to prevent bacteria-laden soil from entering the vasculature. This mechanism likely conferred a survival advantage over organisms lacking such coagulation factors. Thus, we hypothesize that coagulation factors upstream of factor IX evolved primarily to counter foreign substances, particularly pathogenic microbes, entering the bloodstream.

Marine mammals have far fewer opportunities to encounter soil and its associated microbes; over evolutionary time, marine bacterial communities also diverged substantially from those on land [14]. During this transition, many marine mammals gradually lost FXII. In cetaceans, prolonged selection in the deep ocean led to loss-of-function changes—and in some cases functional inactivation—of the F12 gene [15]. The coagulation systems of baleen whales and other deep-diving mammals exhibit a distinctive environmental adaptation: in the high-pressure deep sea, surges in endothelial shear stress could make FXII-mediated contact activation a liability by predisposing to fatal deep-vein thrombosis [16]. Collectively, these observations illustrate a broader theme in coagulation evolution: contact activation likely arose first on land, whereas some mammalian lineages that secondarily returned to the sea later discarded key components of this system as their environments changed.

A review of past research reveals that substances capable of activating FXII are typically foreign materials not naturally present in the blood, such as bacteria or thrombi [17]. The contact activation system has garnered significant attention from ICU physicians because evolutionary insights suggest it serves not only to promote coagulation but also to identify and immobilize foreign substances through thrombus formation. For ICU patients, the introduction of foreign materials into the bloodstream is often unavoidable. Organ failure necessitates support from artificial devices like Continuous Renal Replacement Therapy (CRRT), ECMO, or Intra-aortic Balloon Pump (IABP), all of which involve extensive foreign surfaces in their circuitry. Consequently, anticoagulation strategies for these artificial circuits are a critical focus for ICU clinicians. Similarly, sepsis can be broadly understood as a massive influx of foreign materials—bacteria or bacterial products—triggering a dysregulated systemic inflammatory response. Modulating this immune-inflammatory response remains a key research priority for ICU physicians. Examining the evolutionary role of the contact activation system, it becomes clear that it is not only a safe target for anticoagulation but also a promising novel target for anti-inflammatory interventions. With the development of contact pathway inhibitors, increasing research efforts are focusing on FXII and FXI as potential therapeutic targets.

## 2. Composition of CAS and KKS

CAS and KKS are critical molecular networks regulating coagulation and inflammation. CAS initiates the coagulation cascade via the intrinsic (contact) pathway, comprising coagulation FXII, FXI, plasma prekallikrein (PK), and high-molecular-weight kininogen (HMWK). KKS, in contrast, generates bradykinin through the actions of FXII, PK, HMWK, tissue kallikrein, and low-molecular-weight kininogen (LMWK), mediating inflammation, vasodilation, and tissue repair. Sharing core components (e.g., FXII and HMWK), these systems form a bridge between coagulation and inflammation. This section explores the composition, interactions, and physiological roles of CAS and KKS, focusing on their functions in coagulation, inflammation, and immunothrombosis, with an emphasis on the structural domains and three forms of FXII (zymogen, αFXIIa, and βFXIIa).

### 2.1. Coagulation Factor XII

**Figure 1:**
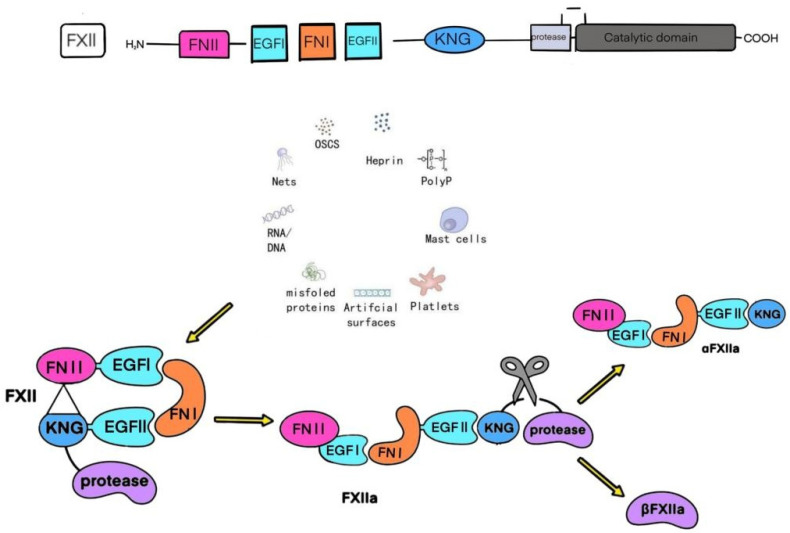
Three Conformational States of Coagulation Factor XII (FXII). Upon exposure to activators—including oversulfated chondroitin sulfate (OSCS), heparin, polyphosphates (polyP), mast cells, platelets, artificial surfaces, misfolded proteins, DNA, or RNA—FXII undergoes conformational transition to αFXIIa. PK subsequently cleaves αFXIIa, generating the low-molecular-weight fragment βFXIIa.

Coagulation factor XII, also known as Hageman factor. It is a serine protease zymogen synthesized primarily by the liver, with a plasma concentration of approximately 40 μg/mL, and is secreted in small amounts by neutrophils [18]. As illustrated in Figure 1, FXII functions through three forms: 1. FXII zymogen: An inactive, closed conformation that serves as a signaling molecule, binding to receptors such as uPAR [19]. 2. αFXIIa: Activated by cleavage on negatively charged surfaces (e.g., kaolin or bacterial surfaces) or by kallikrein, retaining both heavy and light chains. It remains surface-bound, activating FXI and PK [20]. 3. βFXIIa: Formed by further cleavage of αFXIIa by kallikrein, losing the heavy chain (approximately 28–30 kDa) and becoming soluble, primarily activating PK [21]. The fibronectin type I domain of FXII recognizes negatively charged surfaces, such as silicates, exposed DNA, or misfolded proteins, triggering conformational changes and enzymatic activation [22]. This property positions FXII as a shared initiator of CAS and KKS, orchestrating both coagulation and inflammatory responses.

### 2.2. The FXII–αFXIIa–FXIa Axis

The understanding of FXII originated from its role in the contact activation pathway. FXII binds to various artificial surfaces (e.g., glass or plastic) through its fibronectin type I domain, undergoing a conformational change from a closed to an open state, which leads to its activation into αFXIIa [22]. At the adsorbed interface, αFXIIa activates FXI into FXIa, initiating the intrinsic coagulation pathway, and cleaves PK into kallikrein (PKa). Concurrently, PKa further cleaves αFXIIa, generating a low-molecular-weight fragment, βFXIIa [20]. Lacking the heavy chain domain, βFXIIa dissociates from the activation surface into the fluid phase, where it interacts with plasma prekallikrein, cleaving it into active kallikrein (PKa) [21]. PKa sustains FXII activation through a positive feedback loop, creating a feedforward cycle that amplifies FXII activation and produces additional αFXIIa [21]. αFXIIa, anchored to the activation surface, amplifies the coagulation response by activating FXI. Beyond artificial surfaces, FXII can also be activated and adhere to biological entities such as bacteria, misfolded proteins, and exposed DNA [23]. This suggests that the contact pathway may be triggered by pathogenic patterns or aberrant self-components, thereby activating the coagulation system.

### 2.3. The FXII–βFXIIa–KKS Axis

**Figure 2:**
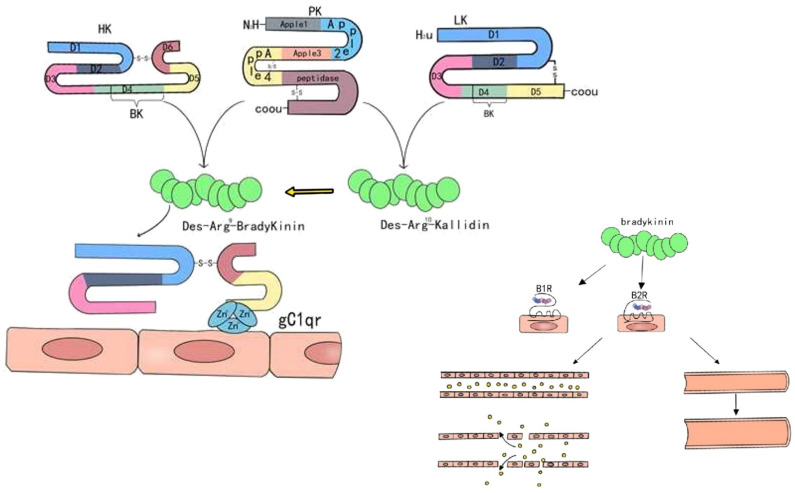
HMWK is hydrolyzed by PK, leading to the cleavage of the BK sequence within its D4 domain and the subsequent release of bradykinin. In contrast, low-molecular-weight kininogen (LMWK) is hydrolyzed by tyrosine kinase (TK), similarly resulting in the release of the bradykinin sequence from its D4 domain. Unlike HMWK, the product released from LMWK is a decapeptide called des-Arg-bradykinin (KD LysBK). Under the action of aminopeptidases, this peptide loses its lysine residue and is converted into bradykinin. Bradykinin exerts its effects by acting on B1R and B2R, leading to edema, vasodilation, and extravasation of tissue fluid. Because B1R are minimally expressed on the cell membrane under normal conditions, the acute-phase effects of bradykinin are primarily mediated through B2 receptors. However, during the chronic phase, B1R expression increases, and both receptor types become involved in the process.

FXII also plays a pivotal role in activating KKS [24]. As described in the FXII–αFXIIa–FXIa axis, the low-molecular-weight βFXIIa, generated in the fluid phase, activates prekallikrein (PKa), which in turn cleaves high-molecular-weight kininogen (HMWK) to continuously produce bradykinin [21]. HMWK is a single-chain glycoprotein with a molecular weight of approximately 120 kDa, comprising 626 amino acids, and is critically involved in physiological processes such as coagulation initiation, complement activation, and inflammation [25]. Studies indicate that HMWK inhibits cell adhesion and angiogenesis and may have potential in antitumor therapy. HMWK consists of six functional domains (D1–D6) [25]: D1–D3 are cysteine-rich, binding calcium ions and mediating protein interactions; D4 contains the bradykinin sequence, which is released upon cleavage; D5 binds to negatively charged surfaces, enhancing contact activation; and D6 interacts with prekallikrein. Plasma kallikrein cleaves HMWK at the D4 domain, releasing a nine-amino-acid peptide known as bradykinin [26]. BK acts on two receptors: Bradykinin B1 receptor (B1R) and Bradykinin B2 receptor (B2R). B1R is expressed at very low levels under normal conditions, while B2R is constitutively expressed on cell surfaces [27]. In endothelial cells, BK binding to B2R increases intracellular Ca^2+^ levels, activating nitric oxide synthase, which leads to the production of nitric oxide and prostaglandins, ultimately causing vasodilation [28]. B1R expression is rapidly upregulated following tissue injury or inflammation [29]. Inflammatory mediators such as Interleukin-1 beta (IL-1β) and Tumor Necrosis Factor (TNF) can induce B1R expression by activating transcription factors like nuclear factor kappa-light-chain-enhancer of activated B cells (NF-κ),activator protein 1(AP-1) and cAMP response element-binding protein (CREB), which bind to the B1R promoter [30,31,32,33]. Activation of B1R by kinins on endothelial cells and neutrophils plays a significant role in the accumulation and activation of neutrophils under inflammatory conditions [34]. As shown in Figure 2, high-molecular-weight and low-molecular-weight kininogens are hydrolyzed to produce bradykinin, which then acts on bradykinin receptors. Upon binding to B2R, BK triggers pro-inflammatory signaling pathways, leading to vasodilation, neutrophil chemotaxis, and increased vascular permeability [35,36,37], resulting in redness, heat, and swelling.

## 3. Significance of CAS in Sepsis

The role of CAS in sepsis is best understood in conjunction with KKS. FXII serves as a sentinel in terrestrial mammals, facilitating the elimination of aberrant self-components and combating infections through three distinct forms. The significance of CAS in sepsis can be elucidated from four key perspectives: 1. Role of FXII in Immunothrombosis: FXII, in its zymogen form, plays a pivotal role in the formation of immunothrombi. By interacting with uPAR on damaged cells, FXII not only influences neutrophils but also affects granulocytes, promoting chemotaxis, localization, and the generation of NETs. These actions establish FXII as a critical initiator of immunothrombosis. 2. Activation of KKS by αFXIIa and βFXIIa: Both αFXIIa and βFXIIa contribute to the activation of the KKS, which can precipitate systemic symptoms such as severe hypotension and profound tissue edema. This activation amplifies the systemic inflammatory response, exacerbating the clinical manifestations of sepsis. 3. KKS Activation and Pathogen Dissemination: Activation of the KKS creates favorable conditions for pathogen survival and dissemination. Pathogenic microorganisms exploit KKS activation to enhance vascular permeability, allowing their infiltration into interstitial tissues and causing organ-specific pathology. Furthermore, KKS activation leads to the production of BK, which upregulates BK receptor expression. This, in turn, increases the likelihood of pathogens entering host cells via these receptors. 4. KKS Storm and Tissue Damage: Excessive KKS activation, often termed a “KKS storm,” contributes to tissue injury and interstitial edema. Modulating the activity of various protein components within the KKS can mitigate the deleterious effects of bradykinin on target organs, thereby reducing tissue damage. 

### 3.1. FXII and Immunothrombosis: The FXII–uPAR Axis

**Figure 3:**
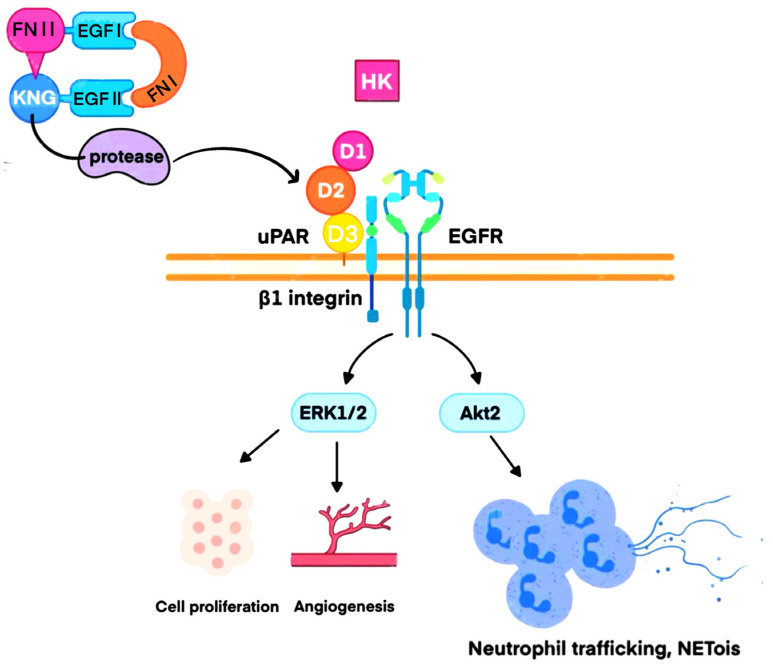
Autocrine FXII binds to uPAR on the surface of neutrophils in a zinc-dependent (Zn^2+^) manner. Signaling assays demonstrate that the interaction between FXII and uPAR promotes phosphorylation of Akt2, leading to extracellular DNA release and the formation of NETs. In addition, FXII can stimulate ERK1/2 phosphorylation via uPAR, specific integrins, and the epidermal growth factor receptor (EGFR), thereby promoting endothelial cell proliferation, growth, and angiogenesis.

FXII circulates in the bloodstream in a closed zymogen conformation, yet even in this inactive form, it plays a pivotal role in signal transduction pathways [38]. As illustrated in Figure 3, FXII can bind to uPAR on the cell surface, thereby exerting a range of physiological effects. uPAR is a glycosylphosphatidylinositol (GPI)-anchored membrane receptor that is widely expressed on various cell types, including endothelial cells, monocytes, and neutrophils [39]. The interaction between FXII and uPAR is primarily mediated through the fibronectin type II domain of FXII and the second domain of uPAR [40]. Importantly, this binding does not require the enzymatic activity of FXII, but instead relies on its zymogen form to initiate signaling. The formation of the FXII–uPAR receptor complex activates several downstream signaling cascades: Activation of the Akt pathway [41]. Phosphorylation of Akt promotes cell survival and metabolism while inhibiting apoptosis [42]. In diabetic nephropathy, FXII-induced activation of the Akt signaling pathway has been closely linked to tubular cell senescence [38]. Activation of the ERK1/2 pathway [41]. FXII binding to uPAR leads to MEK1 activation, which in turn phosphorylates ERK1/2, thereby promoting cellular proliferation and migration [41]. Activation of the integrin β1 signaling pathway. The FXII–uPAR complex also interacts with integrin β1, triggering the downstream PI3K signaling cascade, which regulates cell adhesion and migration [38].

Experiments have revealed that, besides hepatocytes, neutrophils themselves can secrete small amounts of FXII through an autocrine mechanism. This locally produced FXII acts on the neutrophil’s own uPAR to exert biological effects [43,44]. Crucially, one of the most important of these effects is the formation of NETs. NETs represent a novel antimicrobial strategy employed by neutrophils [45]. They are web-like meshworks composed of a DNA backbone studded with antimicrobial proteins, including histones, myeloperoxidase (MPO), and neutrophil elastase (NE) [45]. Neutrophils actively participate in Disseminated Intravascular Coagulation (DIC) associated with thrombosis, specifically by promoting contact pathway activity and subsequent thrombin generation through NETs [46]. The cell-free DNA within NETs provides the negatively charged surface necessary to activate coagulation factor XII, thereby mediating thrombin production [47]. Furthermore, NETs formed after neutrophil activation serve as scaffolds upon which thrombi develop [48,49]. FXII triggers NETosis by interacting with the D2 domain of uPAR on the neutrophil surface, leading to phosphorylation of Akt2 and initiation of the NET release process [50,51]. Through these pathways, FXII and NETs significantly potentiate the formation of immunothrombi. Remarkably, the neutrophil surface can even immobilize components of CAS, providing a solid-phase platform for the sustained activation of both the CAS and KKS. This establishes the neutrophil as a central driver—effectively an ‘engine’—for immunothrombosis [43]. Thrombosis driven by neutrophil extracellular traps (NETs) is not confined to the contact activation system. NET constituents—the negatively charged DNA backbone, histones H3/H4, neutrophil elastase (NE), myeloperoxidase (MPO), and cathelicidin—promote thrombosis through multiple pathways: they induce von Willebrand factor (vWF)–platelet string formation, activate and aggregate platelets, enhance thrombin generation and tissue factor (TF) release, and concurrently suppress fibrinolysis [52]. Accordingly, inhibiting factor XII (FXII) alone is unlikely to adequately modulate NET-mediated immunothrombosis. The relationship between FXII and NETs merits further investigation.

### 3.2. Hereditary Angioedema and the Kallikrein-Kinin System (KKS) Storm

Hereditary angioedema (HAE) is a bradykinin-mediated angioedema primarily caused by C1 inhibitor (C1-INH) deficiency or dysfunction, leading to excessive activation of Factor XII [53]. It is characterized by recurrent, acute episodes of non-pitting, localized swelling affecting subcutaneous and/or submucosal tissues. Common sites include the face, extremities, trunk, genitalia, upper airway, and gastrointestinal tract [54]. HAE is predominantly classified into two types: Type I, characterized by C1-INH deficiency, and Type II, characterized by dysfunctional C1-INH. C1-INH normally regulates proteases within the contact system, including PK, FXIIa, and FXIa [55]. In HAE patients, the functional loss of C1-INH results in uncontrolled and sustained activation of the contact and complement systems. This dysregulation leads to continuous kallikrein-mediated bradykinin generation [56,57]. BK increases vascular permeability, causing edema formation. The pathophysiology and clinical manifestations of HAE provide a direct illustration of the inflammatory edema resulting from a “KKS storm.” A strikingly similar pathological cascade, involving KKS dysregulation and inflammatory injury, was observed in severe Coronavirus Disease 2019 (COVID-19).

The spike protein (S protein) on the surface of Severe Acute Respiratory Syndrome Coronavirus 2 (SARS-CoV-2) directly binds to the Angiotensin-converting enzyme 2 (ACE2) receptor, triggering membrane fusion or endocytosis to enable viral entry into host cells [58]. Following infection, ACE2 receptors are internalized with the virus, significantly reducing their expression on the cell surface [58]. Additionally, viral infection may involve proteases like Transmembrane protease, serine 2 (TMPRSS2) cleaving ACE2, further impairing its function [59]. ACE2 normally serves two key physiological roles: (1) it degrades angiotensin II (AngII) into anti-inflammatory Ang(1-7), maintaining vascular homeostasis [60]; and (2) it indirectly regulates bradykinin levels by balancing the renin-angiotensin system (RAS), though it is less effective than angiotensin-converting enzyme (ACE) at directly degrading bradykinin [61]. Reduced ACE2 expression leads to AngII accumulation, activating the AT1 receptor and inducing vasoconstriction, oxidative stress, and pro-inflammatory cytokine release [62,63]. Impaired ACE2 function disrupts RAS regulation, indirectly reducing ACE activity (a key bradykinin-degrading enzyme), resulting in bradykinin accumulation. Bradykinin, acting through the B2R, increases vascular permeability, leading to pulmonary capillary leakage, worsening hypoxemia, and causing pulmonary edema, which manifests radiologically as “ground-glass opacities.” This amplifies inflammation, promoting monocyte infiltration and the release of pro-inflammatory cytokines like IL-6 and TNF-α, driving a “cytokine storm.” It also activates pain receptors, exacerbating dyspnea and systemic symptoms [64]. Clinically, dry cough, one of the most common symptoms, may be attributed to increased bradykinin activity [65]. Reduced oxygen saturation, bronchospasm, and vascular leakage can also be explained by elevated bradykinin levels. Excessive bradykinin may cause hypokalemia, potentially leading to arrhythmias and sudden death [65]. Based on this, 2020 case reports suggested that icatibant, a B2R antagonist, might improve pulmonary edema in COVID-19 patients, though these studies had small sample sizes [66,67]. Some clinical trials explored C1-INH to mitigate the cytokine storm, with results showing improved oxygenation in some patients [68]. However, with the end of the COVID-19 pandemic, most of these trials were discontinued. Garadacimab, an FXII inhibitor, showed no clear benefit in COVID-19 treatment [69]. Thus, neither FXII inhibitors nor other KKS inhibitors have demonstrated consistent benefits for sepsis or related conditions based on available clinical and animal data. Beyond causing damage through a KKS-driven “storm,” the KKS can also be exploited by bacteria to facilitate colonization in humans and higher animals.

### 3.3. Pathogen Exploitation of KKS Activation Facilitates Virulence

The local release of bradykinin, acting on vascular endothelial cells, can lead to the leakage of nutrient-rich plasma into tissue spaces, providing a fertile environment for bacterial growth. Consequently, pathogenic microorganisms can exploit CAS to induce BK production, increasing vascular permeability [70]. This not only exacerbates the severity of sepsis but may also promote invasive spread. Current research indicates that not all bacteria in nature possess this capability; only certain bacterial species exhibit these characteristics.

*Staphylococcus aureus* expresses staphylocoagulase and staphylokinase, enhancing FXII activation and fibrin formation [71,72]. The resultant fibrin barrier shields bacteria from phagocytosis while permitting nutrient diffusion [73]. Porphyromonas gingivalis secretes gingipains that directly cleave HMWK to generate BK, exacerbating periodontal inflammation and facilitating bacterial colonization [74,75]. By inducing BK overproduction, bacteria create a hyperinflammatory milieu that overwhelms host defenses, promoting tissue damage and dissemination. This strategy establishes a protective microenvironment enabling evasion of phagocytosis and complement-mediated killing.

*Streptococcus pyogenes* employs more complex mechanisms: its M-protein binds FXII, promoting activation and fibrin deposition to mask bacterial antigens from immune recognition [76]. Concurrent streptokinase secretion enhances PK activation, causing excessive BK generation. The resulting uncontrolled vascular leakage facilitates deep tissue invasion. In severe streptococcal infections, surging BK levels may trigger a “bradykinin storm,” manifesting as hypotension and capillary leak syndrome (e.g., toxic shock syndrome). B2R antagonists have been shown to significantly improve survival in murine models of streptococcal sepsis [77]. Notably, NETs provide electronegative DNA surfaces that accelerate CAS activation [78]. Histones and antimicrobial proteins within NETs can damage vascular endothelium, exposing subendothelial collagen that further activates the extrinsic coagulation cascade (tissue factor pathway) [79], creating a dual-pathway procoagulant effect.

Both *S. aureus* and *S. pyogenes* secrete nucleases that degrade NET DNA, liberating entrapped bacteria while providing additional electronegative surfaces for FXII activation [80,81]. This amplifies the CAS, enhancing thrombin generation and fibrin deposition to reinforce bacterial shielding. Remarkably, neutrophils serve as CAS/KKS activation platforms. Bacteria-triggered neutrophils release FXII, which binds uPAR on neutrophil surfaces, inducing Akt2 phosphorylation and NETosis. *S. aureus* and Pseudomonas aeruginosa exploit this platform to survive within thrombi and evade immune clearance. This self-reinforcing cycle establishes neutrophils as inadvertent “engines” of immunothrombosis, paradoxically promoting bacterial persistence.

### 3.4. FXII Responses to Altered Self: Implications for Extracorporeal Organ Support

The use of invasive medical devices is increasingly common in both general wards and ICUs. Examples include stent implantation for acute myocardial infarction, mechanical valve replacement for heart valves, and organ support in the ICU, such as ECMO, IABP, and CRRT. These intravascular organ support devices all require anticoagulation to prevent clotting. When FXII comes into contact with artificial materials—such as the negatively charged surfaces of percutaneous coronary intervention (PCI) stents, ECMO circuits, or the hollow fiber lumens in CRRT filters—it triggers thrombus formation via the FXIIa-α–FXIIa–FXIa axis. These artificial negatively charged surfaces can be viewed as an extension of “non-self” entities. Consequently, the application of contact pathway inhibitors has become a hot topic in recent years. FXI inhibitors can block the FXIIa-α–FXIIa–FXIa axis, preventing thrombin generation through FXIa and the feedback activation of FXI by thrombin. Numerous drugs have entered clinical trials, but anticoagulation is not the primary focus of this discussion, so it will not be elaborated further here. The key point to address is the FXII-β–FXIIa–KKS axis activated by these artificial negatively charged surfaces. FXII inhibitors not only suppress the FXIIa-α–FXIIa–FXIa axis to prevent thrombosis but also interrupt the FXII-β–FXIIa–KKS axis to avert a KKS storm. Since the main clinical indication for FXII inhibitors is the treatment of hereditary angioedema (HAE), research on their use for anticoagulation and anti-inflammatory effects in artificial circuits has been limited to animal studies.

In rabbit venoarterial ECMO models, antisense oligonucleotide (ASO)-mediated reduction in FXII or FXI significantly prolonged circuit lifespan while reducing fibrinogen consumption. Crucially, neither FXII nor FXI inhibition caused clinically significant bleeding at surgical sites or in critical organs (brain, liver, kidneys). Importantly, FXII suppression demonstrated unique organ-protective effects: FXII-inhibited subjects exhibited substantially reduced pulmonary edema, whereas control groups, heparin-treated cohorts, and FXI-deficient animals sustained notable lung injury [82]. Complementary rat studies revealed that FXII deficiency attenuated neutrophil migration to the liver during ECMO. Elevated cleavage of high-molecular-weight kininogen and complement activation—both hallmark ECMO complications—were abolished by FXII gene knockout [83]. Although these ECMO models and therapies remain untested in humans, preclinical evidence indicates that inhibiting artificial surface-induced activation of the FXII→β-FXIIa→KKS axis during extracorporeal support could mitigate systemic inflammation and facilitate recovery.

### 3.5. Non-Coagulation Functions of Factors in the Contact Activation System

Historically, CAS was thought to function solely within the coagulation cascade, with no independent roles in signal transduction or protein domains unrelated to anticoagulation. However, recent research has overturned this view. FXI, also known as coagulation FXIa, is a serine protease secreted by the liver with a molecular weight of approximately 160 kDa [84]. It exists as a dimer composed of two identical subunits, each containing 607 amino acids linked by disulfide bonds [84]. While FXI was traditionally considered a key enzyme in the coagulation cascade, recent studies reveal that FXIa is not only an amplifier of coagulation but also interacts with platelets and endothelial cells, mediating inflammatory kinin production by activating FXII and cleaving high-molecular-weight kininogen to generate bradykinin. Furthermore, it acts as a signaling molecule capable of “conditioning” various cellular responses [85]. Activation of FXI increases vascular permeability in vivo. FXIa enhances endothelial cell permeability by inducing cleavage of the extracellular domain of vascular endothelial (VE)-cadherin, releasing soluble fragments. In preliminary experiments using a mature baboon sepsis model, inhibiting FXI activation significantly reduced soluble VE-cadherin levels, thereby preserving vascular barrier function [86]. FXIa is not merely an amplifier in the coagulation cascade; it also drives smooth muscle cell migration via the protease-activated receptor 1 (PAR1)–L-type voltage-dependent calcium channel alpha-1C subunit–protein kinase C axis, marking the first evidence of a direct cellular effect of FXIa on smooth muscle cells [87].

FXII has also emerged as a research focus in recent years. Beyond its role as a proenzyme and signaling molecule, FXII and its subunits may exert antibacterial effects during bacterial infections. In a mouse model of disseminated intravascular coagulation (DIC) induced by Pseudomonas aeruginosa, administration of FXII or its heavy chain reduced tissue bacterial load, mitigated organ pathology, decreased fibrin deposition, and improved survival. Studies have shown that FXII and its heavy chain inhibit and kill P. aeruginosa growth, whereas the light chain has no effect. The heavy chain of FXII can hydrolyze lipopolysaccharide (LPS), degrading this critical outer membrane component in a concentration-dependent manner [88].

## 4. Perspectives

The evolutionary trajectory of the coagulation system in higher animals suggests that CAS is primarily geared toward combating microbial invasion and mitigating the harmful effects of “abnormal self” entities within the body. This perspective implies that, in the ICU, where the battle against external microbes is critical, anticoagulation and anti-inflammatory strategies should be considered together. Under the premise of effective anti-infective therapy, the use of contact pathway inhibitors for anticoagulation may simultaneously reduce the intensity of inflammatory responses, thereby alleviating target organ damage caused by systemic inflammation. Numerous compounds targeting the contact pathway have advanced to clinical trials. FXI inhibitors are primarily indicated for preventing lower extremity deep vein thrombosis, while FXII inhibitors are mainly used for treating HAE. Table 1 introduces the contact pathway inhibitors that have entered clinical trials in recent years. Since its discovery, the contact activation system has been a focal point of research. With the development of inhibitors, an increasing number of clinicians and researchers are revisiting this system. Sepsis is a complex disease driven by multiple factors, and the role of the contact activation pathway in its pathophysiology continues to be explored. Contact pathway inhibitors not only offer superior anticoagulation effects compared to heparin in sepsis treatment but also represent a novel target for reducing inflammation-induced damage, warranting further research investment.

## Figures and Tables

**Table 1:** Recent Progress in Clinical Trials of Contact Pathway Inhibitors [89].

Drug Type	Representative Drug	Development Stage (as of 2022, Projected to 2025)	Developer	Main Applications (Thrombosis/Sepsis-Related)	Key Findings (2015–2022)
Antisense Oligonucleotide (ASO)	Fitusiran (AT977)	Phase II completed, Phase III ongoing	Sanofi/IONIS	AF stroke prevention, VTE, sickle cell disease stroke	Liver-targeted FXI knockdown, reduced VTE by 70%, low bleeding risk; Phase II trials since 2017.
Antisense Oligonucleotide (ASO)	IONIS-FXI Rx	Phase II completed	IONIS	Post-surgical VTE, ESRD dialysis thrombosis	2015 knee replacement trial: VTE reduced to 4–27% (vs. 30% enoxaparin), no severe bleeding.
Small Molecule Inhibitor	Asundexian (BAY 2433334)	Phase II completed, Phase III initiated (OCEANIC)	Bayer	Stroke/AF prevention, post-MI thrombosis, potential sepsis inflammation	2022 PACIFIC trial: 30% stroke risk reduction, 3–4% bleeding (vs. 2% placebo); Phase III recruiting to 2025.
Small Molecule Inhibitor	Milvexian (JNJ-70033093)	Phase II completed, Phase III planned	Janssen/BMS	Stroke/VTE prevention, cancer-associated thrombosis, sepsis DIC	2021 AXIOMATIC trial: VTE reduced to 8–21% (vs. 21% enoxaparin), moderate bleeding at high doses.
Monoclonal Antibody	Abelacimab (MAA868)	Phase II completed, Phase III recruiting (MAGNOLIA/ASTER)	Anthos Therapeutics	AF stroke, cancer VTE, ECMO-related thrombosis	2021 knee trial: VTE 4–13% (vs. 22% enoxaparin); Phase III vs. dabigatran to 2025.
Monoclonal Antibody	Osocimab (BAY 1213790)	Phase II completed	Bayer	Surgical VTE, ESRD dialysis	2020 FOXTROT trial: superior to enoxaparin, low bleeding; 2022 ESRD trial results pending.
Other (Aptamer)	FELIAP	Preclinical	Research Institutions	Potential reversible FXIa inhibition, sepsis/inflammatory thrombosis	In vitro FXIa-FIX activation suppression; no clinical advancement post-2015, needs reversal agents.

## Data Availability

No new data were created or analyzed in this study.
